# Beyond temperature coupling: Effects of temperature on ectotherm signaling and mate choice and the implications for communication in multispecies assemblages

**DOI:** 10.1002/ece3.3059

**Published:** 2017-06-28

**Authors:** Laurel B. Symes, Rafael L. Rodríguez, Gerlinde Höbel

**Affiliations:** ^1^ Department of Biological Sciences University of Wisconsin Milwaukee WI USA; ^2^ Department of Biological Sciences Dartmouth College Hanover NH USA

**Keywords:** character displacement, reproductive interference

## Abstract

Many organisms share communication channels, generating complex signaling environments that increase the risk of signal interference. Variation in abiotic conditions, such as temperature, may further exacerbate signal interference, particularly in ectotherms. We tested the effects of temperature on the pulse rate of male signals in a community of *Oecanthus* tree crickets, and for one focal species we also assessed its effect on female pulse rate preferences and motivation to seek mates. We confirm prior findings of temperature‐dependent signals that result in increasing signal similarity at lower temperatures. Temperature also affected several aspects of female preferences: The preferred pulse rate value was temperature dependent, and nearly perfectly coupled with signal pulse rate; the range of pulse rate values that females found attractive also increased with temperature. By contrast, the motivation of females to perform phonotaxis was unaffected by temperature. Thus, at lower temperatures the signals of closely related species were more similar and females more discriminating. However, because signal similarity increased more strongly than female discrimination, signal interference and the likelihood of mismating may increase as temperatures drop. We suggest that a community approach will be useful for understanding the role of environmental variability in the evolution of communication systems.

## 1 INTRODUCTION

There are relatively few sensory channels and many organisms that use them for communication (Bradbury & Vehrencamp, 1998; Greenfield, 2002). When many animals communicate on the same channel at a given place and time, they face problems arising from signal overlap and interference. Consider, for example, a frog or a katydid attempting to find a mate at a multispecies chorus—a spectacular riot of signals produced by individuals ranging in suitability as mates from attractive and unattractive conspecifics to wholly unacceptable heterospecifics (Gerhardt & Huber, 2002; Greenfield, 2002). The greater the number of species using the same communication channel and the greater the similarity in their signals, the greater the risk that their signals will mask each other, decreasing their effectiveness and range, and increasing the likelihood of mismating (Brumm 2013; Gerhardt & Huber, 2002; Greenfield, 2002).

Environmental conditions can affect the nature and severity of the problems that result from many organisms sharing the same communication space. For instance, the signals and receiver responses of ectotherms such as fish, frogs, and insects change with ambient temperature (Doherty, 1985; Gayou, 1984; Gerhardt, 1978; Gerhardt & Huber, 2002; Grace & Shaw, 2011; Papes & Ladich, 2011; Pires & Hoy, 1992). Temperature‐dependent changes in signals often correspond to changes in mate preferences; that is, there often is signal‐preference temperature coupling (Doherty, 1985; Gerhardt, 1978; Jang & Gerhardt, 2006; Pires & Hoy, 1992; Walker, 1957, 1962, 1963). However, the degree of signal‐preference correspondence may vary all the way from close to loose or absent (Doherty, 1985; Gerhardt & Mudry, 1980; Ritchie, Saarikettu, Livingstone, & Hoikkala, 2001; Skovmand & Pedersen, 1983), or it may take a form that does not promote assortative mating (Greenfield & Medlock 2007). Additionally, the rate of change in signals with temperature varies among species, with the consequence that not only the signal itself, but also the degree of similarity between the signals of different species, varies with temperature (Jang & Gerhardt, 2006; Walker, 1957, 1962, 1963).

**Figure 1:**
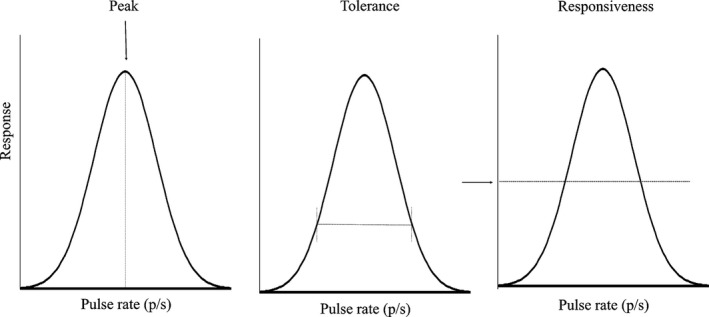
A preference function and the parameters used to describe variation in the function. Peak is the value of the male trait where the response was highest. Tolerance is measured as the breadth of the function at 1/3 of the peak height as a standardized way of estimating the range of male traits that elicit response. Responsiveness is the mean height of the preference function across all stimuli for which the female was tested

**Figure 2:**
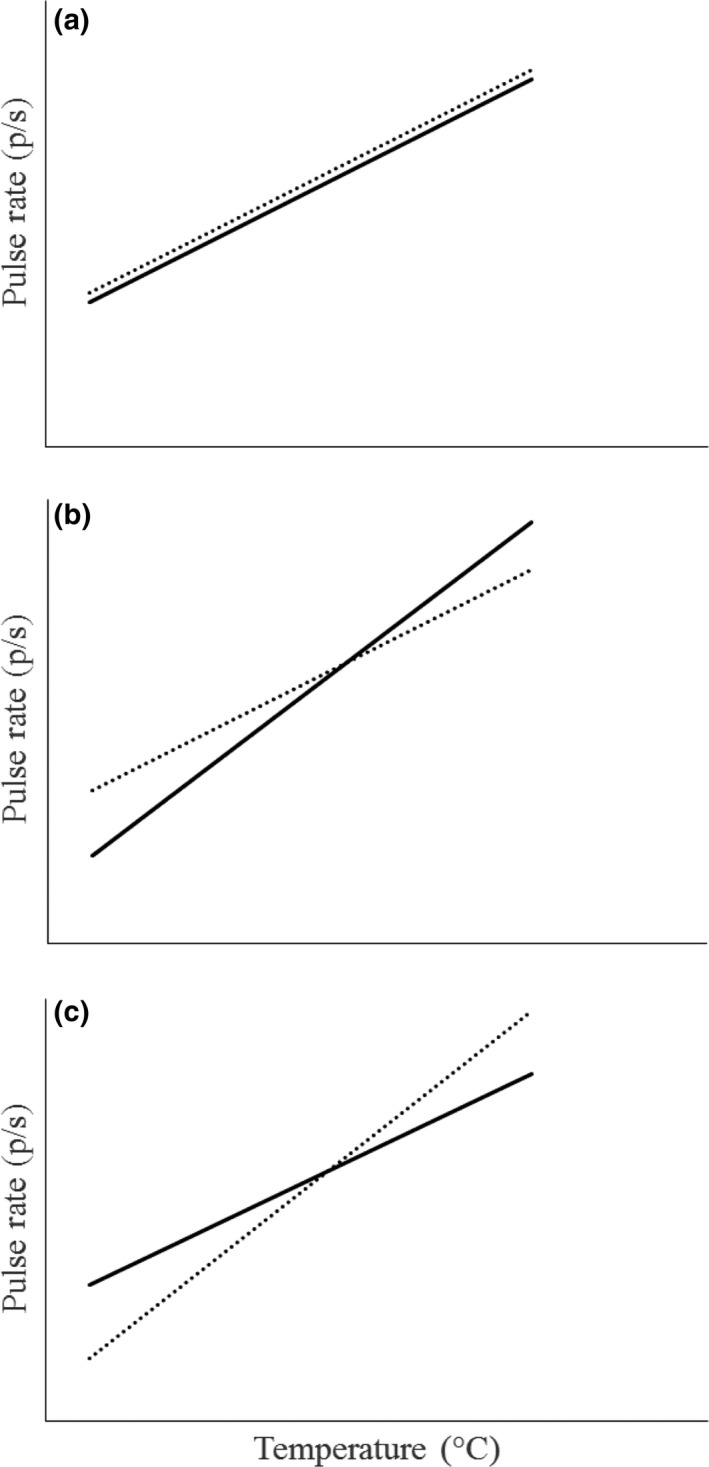
Hypothetical effects of temperature on the relationship between male pulse rate (solid line) and female peak preference (dotted line). Males and females may show analogous temperature coupling (a), greater temperature dependence of male signals (b), or greater temperature dependence of female preference peak (c)

**Figure 3:**
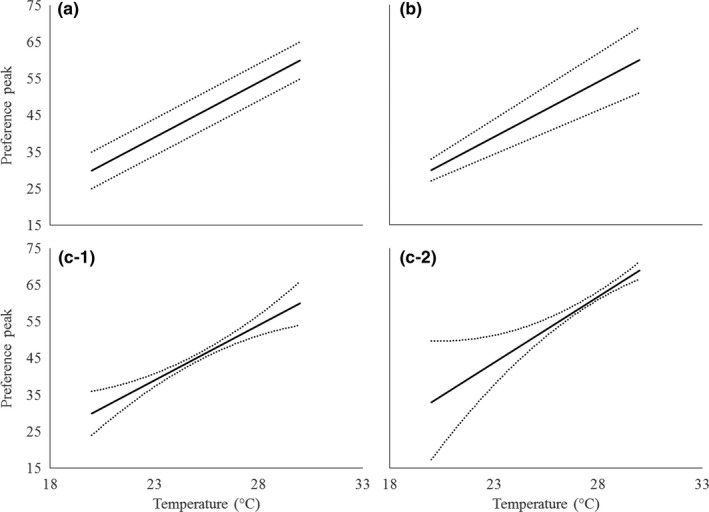
Potential relationships between temperature and tolerance in female *Oecanthus* crickets. Solid lines show female peak preference, and dotted lines show the upper and lower limits of the pulse rate that elicit female response (tolerance). Panel a shows constant tolerance. Panel b shows tolerance that scales with the magnitude of the signal. Panels c‐1 and c‐2 show two samples of minimum tolerance at a particular temperature

The amount of signal interference generated by environmental variation may depend on how different species in the community respond to environmental variation (Hebets et al., 2015; Leal & Losos, 2015). This is a broad question that can involve any and all species using a given sensory channel in a given environment. However, it may be possible to advance by focusing on closely related species, for which the likelihood of signal similarity and interference and the risk and negative consequences of mismating may be particularly high. Here, we test the hypothesis that ambient temperature influences the likelihood of interspecific signal discrimination between closely related species. This hypothesis makes the following two predictions: (1) The similarity of the signals of different species within a community will vary with temperature in a way that influences the likelihood of signal interference. This prediction has been supported in prior work (Jang & Gerhardt, 2006; Walker, 1957, 1962, 1963), and here, we confirm it for our study species (see below). (2) The signal‐preference relationship will vary with temperature in a way that influences discrimination of heterospecifics signals. To test this second prediction, it is important to test multiple attributes of mate preferences, any one of which may influence patterns of mating and the likelihood of assortative mating, the extent of reproductive interference, and the strength or directionality of selection that female preference imposes on male traits. There may be variation in the preferred signal trait value (“peak preference”), the range of signal values that elicit a given level of response (“preference tolerance”), and the overall level of response across signal values (“responsiveness”) (Figure 1) (Bailey, 2008; Fowler‐Finn & Rodríguez, 2012; Ritchie et al., 2001; Rodríguez et al. 2013). Most research on temperature coupling has focused on peak preferences (Gerhardt, 1978; Grace & Shaw, 2004; Ritchie et al., 2001), but there is evidence suggesting that temperature does influence tolerance (Ritchie et al., 2001). Prediction (2) may involve a variety of scenarios. Across temperatures, peak preference may track the change in signals (perfect temperature coupling, whereby selection due to mate choice would remain consistent across temperatures), or change more or less steeply (imperfect coupling that may impose temperature‐dependent directional selection) (Figure 2). Similarly, tolerance may remain constant, increase, or decrease with temperature, which could lead to changes in the strength of selection (Figure 3). It may seem likely that tolerance will increase with temperature—consider that, as signaling rates increase with temperature, discriminating between signals would require greater relative differences, in accordance with Weber's law (Bradbury & Vehrencamp, 1998). However, the effect of temperature on tolerance may depend on the extent to which receivers are adapted to preserve patterns of mate choice across temperatures (Greenfield & Medlock 2007). Consequently, tolerance may be lowest at temperatures where the most species with similar signals are active, when discrimination would be most critical, with tradeoffs in acuity at other temperatures (Stiebler & Narins, 1990) (Figure 3c). Finally, responsiveness may also change with temperature (e.g., for ectotherms one would expect increased responsiveness at higher temperatures), so that most mating happens at warm temperatures, with the result that the form of the signal‐preference relationship at some temperatures makes more important contributions than at other temperatures. Besides describing responsiveness as a feature of mate preferences, we also use two measures of females’ overall motivation to search for mates to aid interpretation of our results.

**Figure 4:**
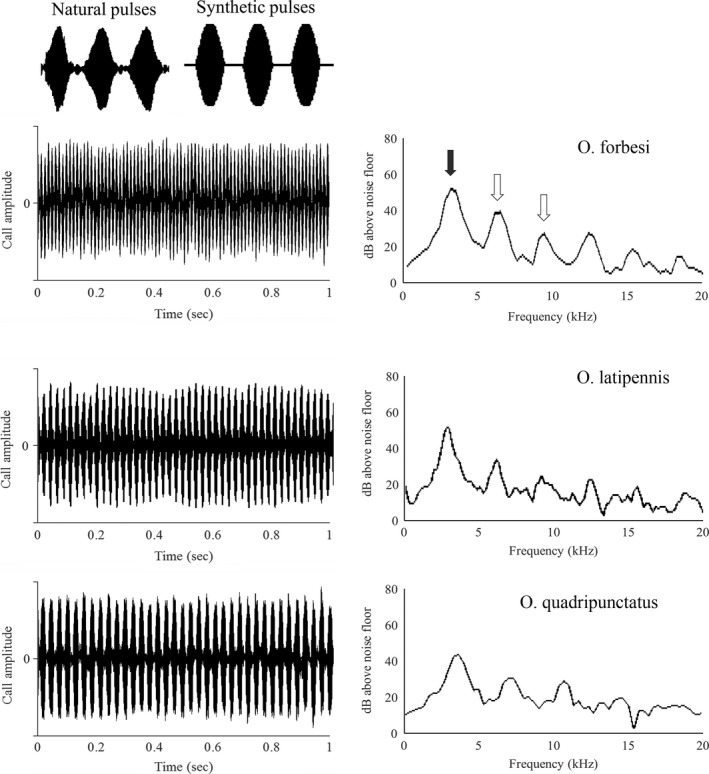
Signal structure of *Oecanthus* signals. The acoustic signal consists of a continuous series of sound pulses, each of which corresponds to one wing closure (left panel, natural pulses and synthetic stimuli). When tree crickets close their wings, they strike teeth in their stridulatory file against a hardened vein on the other wing, producing a pulse of sound. The vibration pattern of the wing membrane determines the fundamental frequency of the signal. The majority of the signal energy is found in a few frequency bands (right panel), with a fundamental frequency that is typically between 3 and 4.5 kHz (filled arrow) and a small amount of energy in the higher harmonics of the fundamental (unfilled arrows)

Here, we test the above predictions by assessing how temperature affects the rate of change in signals and mate preferences. Our study animals were *Oecanthus* tree crickets (Orthoptera: Gryllidae: Oecanthinae). Multiple species of tree cricket often signal and form pairs at the same site. Tree crickets are active across a range of temperatures and have signals that are strongly temperature dependent (Walker, 1962, 1963; Walker & Moore, 2013). Their acoustic signal consists of a series of rapidly repeated pulses, with each wing closure producing one pulse of sound (see Figure 4 for an extended description of sound production). In tree crickets, pulse rate is a primary feature differentiating species (Symes, 2013, 2014; Walker, 1957, 1963). Dominant frequency and pulse duration also vary with temperature in ways that are likely important to female preference (Brown, Wideman, Andrade, Mason, & Gwynne, 1996; Mhatre, Bhattacharya, Robert, & Balakrishnan, 2011). Although females may display preferences for pulse duration and fundamental frequency (Brown et al., 1996), they will respond to signals with heterospecific pulse durations and fundamental frequencies as long as the pulse rate is within their response window (Symes, 2013). Therefore, the current work focuses primarily on how temperature affects female pulse rate preference and the similarity of heterospecific pulse rates.

Like many other ectothermic species, tree cricket pulse rates increase with temperature, but the rate at which pulse rates increase with increasing temperature varies by species (Walker, 1962, 1963). Species with relatively fast pulse rates at a given temperature generally increase in pulse rate more rapidly with temperature (Walker, 1957, 1962, 1963). The result of this rate‐dependent variation in pulse rate increase is that at cool temperatures, the pulse rates of multiple species converge on similar slow values, while at warm temperatures, the species in a community are spread out over a greater range of pulse rates (Walker, 1957). For example, at cool temperatures (such as 15°C), the signal difference between the co‐occurring species *O. forbesi* and *O. latipennis* is 6 pulses/s, while the same two species are separated by 18 pulses/s at 30°C (Walker, 1957). The fact that signal similarity varies with temperature supports the first prediction of the hypothesis that temperature affects the discrimination of signals. We therefore confirm prior support for the first prediction before proceeding to focus on the second prediction, that temperature affects the signal‐preference relationship.

## 2 METHODS

We selected a study area where there are three co‐occurring *Oecanthus* species that produce continuous signals consisting of repeated pulses: *O. forbesi*,* O. latipennis*, and *O. quadripunctatus* (Figure 4). These species are common in secondary growth and weedy areas, and multiple species can be found in the same plant or within hearing distance of other species. We focused on continuously signaling *Oecanthus* species in order to sample the most similar heterospecifics. We note that in the same environment there are also chirping *(O. fultoni)* and trilling (*O. niveus*,* O. exclamationis*) congeners, as well as a suite of more distant acoustically communicating taxa. A broader community focus would provide a more complete understanding in terms of signal interference, but perhaps with lower applicability to the risk of mismating.

### 2.1 Male acoustic signals

All male crickets were collected and recorded between July and October 2012–2014. We collected male crickets from Licking County, OH, USA [39.988, −82.412] (*N*_forbesi_ = 37, *N*_quadripunctatus_ = 3), Greene County, OH, USA [39.754, −83.810] (*N*_forbesi_ = 15, *N*_quadripunctatus_ = 27), Stark County, OH, USA [40.844, 81.441] (*N*_forbesi_ = 6, *N*_latipennis_ = 12, *N*_quadripunctatus_ = 35), and Summit County, OH, USA [40.938, 81.645] (*N*_forbesi_ = 8, *N*_quadripunctatus_ = 1). For the male signal community, samples were pooled across site because previous work in this region has shown rapid dispersal and little geographic variation in tree cricket pulse rate (Symes, 2014; Walker, 1962, 1963).

To quantify how male signals characteristics change with temperature, we analyzed previously published male song data (Symes, 2014) (*N* = 145), supplemented with recordings from additional males (*N* = 12) that provided data of mid‐range and warm temperatures for the focal species *O. forbesi*. Available recordings of heterospecifics from the focal sites spanned a relatively narrow range, but heterospecific males call across a temperature range that equals or exceeds the range of *O. forbesi* (Walker, 1963). Recording equipment, recording conditions, and analysis procedure were the same for all male recordings. In brief, males were recorded under screen tents using a Marantz 661 solid‐state recorder (Mahwah, NJ, USA) at 96 kHz and 24 bit depth. The recorder was connected to a Sennheiser ME 62 microphone with a K6 power module (Solrød Strand, Denmark) housed in a 43.2‐cm Telinga parabolic dish (Tobo, Sweden). The distance from the microphone to the insect was 0.8–1.2 m. Temperature at the time of measurement was recorded using a DT‐172 thermometer placed next to the male (CEM, Shenzhen, China). We analyzed the recordings using Raven Pro version 1.4 (Cornell Lab of Ornithology, Ithaca, New York, USA). Pulse rate was calculated using the interactive detector feature to detect and number individual pulses. Detector parameters were “duration between 0.0029 and 0.0203 s; frequency between 2,000 and 5,000 Hz; minimum separation of 0.0029 s.” We determined how many pulses were produced in 2 s of continuous signaling and divided this value into half to obtain the number of pulses produced per second. Male recordings and metadata are archived at the Macaulay Library of Natural Sound (Cornell University).

In captivity, crickets were fed an ad libitum diet of Fluker's cricket chow (Port Allen, LA, USA) and housed in plastic containers that were 8 cm high and 12 cm in diameter. Each container had a screen lid and a piece of plastic plant for structure.

### 2.2 Female mate preferences

To examine the signal‐preference relationship and how it is influenced by temperature, we focused on one locally abundant species, *O. forbesi*. We collected female *O. forbesi* in Greene County, OH [39.754, −83.810], in late July 2013 and maintained them in the same way as males (see above). Females were collected as juveniles and held singly, ensuring virginity and standardizing mating motivation.

We adopted a function‐valued approach to describe variation in the parameters of female mate preferences (Bailey, 2008; Fowler‐Finn & Rodríguez, 2013; Ritchie et al., 2001). This approach views the trait of interest as the entire curve that describes signal attractiveness as a function of signal trait values. Using synthetic male signals, we measured female preference for a wide range of male pulse rate values and used the responses of the females to construct a preference function that indicates the relative attractiveness of each value of the male trait (Bailey, 2008; Fowler‐Finn & Rodríguez, 2013; Ritchie et al., 2001). The function itself is the female trait under consideration and can then be further quantified with a number of parameters (peak, tolerance, and responsiveness; Figure 1).

To generate female preference functions, we tested female response to a series of synthetic male signals and replicated these measurements at three temperatures where mating commonly occurs (20, 25, and 30°C) (L. Symes, personal observation). The stimulus set consisted of 11 synthetic signals that ranged from 40 to 90 pulses/s in increments of 5 pulses/s. Each female experienced 33 playbacks (11 stimuli × 3 temperatures) over 6–8 days to minimize the potential for fatigue or habituation. Playbacks were organized into sessions with up to three playbacks per session and up to two sessions per day. Sessions were separated by a minimum of an hour. All playbacks in a session were done at the same temperature, but the individuals and sessions were haphazardly allocated among temperatures until all playbacks had been completed. Within session, the three stimuli were randomly selected without replacement from the 11 options until all stimuli had been assigned to a session.

Stimuli were synthesized by adding sine waves using R software and the Sound and Seewave packages (code available on request from authors) (R Core Team 2015; Sueur, Aubin, & Simonis, 2008). Pulses increased in amplitude for the first 45% of the pulse and decreased in amplitude for the last 45% (Figure 4), parameters that are consistent with measurements from males.

Trials were conducted in a semi‐anechoic chamber. The preference arena consisted of a one‐meter ring constructed from noise‐absorbing foam. A 6010A speaker (Genelec, Iisalmi, Finland) was embedded in the wall of the ring. Amplitude was set by playing the population mean stimulus and adjusting the amplitude until an Extech 407764 sound level meter registered 68 dB SPL at the center of the arena. This is comparable to the amplitude of a male signal at the same distance (50 cm) measured with the same instrument. During testing, the chamber was darkened and observed with a red headlight to minimize disturbance to the largely nocturnal animals. Crickets were maintained on a reverse light cycle, and tests were conducted within 5 hr of darkness.

To determine if females were sexually receptive, we first tested them with a stimulus matching the mean signal from their source population. Preliminary investigations showed that females that failed to respond to a population mean signal seldom responded to any other stimuli. Females that responded to the population mean were used in the full set of trials. Females that did not respond to the population mean stimulus the first time they were tested were retested 3–7 days later. Fewer than 10% of the females did not respond to either playback and were not used in the experiment.

At the start of each trial, the female was placed in the center of the arena under a plastic cup. The cup was lifted once stimulus playback was initiated and females were observed to determine whether they made contact with the speaker within 40 s. If the female did not respond within 40 s, we continued the playback for an additional 80 s and recorded whether females made contact with the speaker during this time. Continuing to monitor response for the additional 80 s allowed us to detect whether any temperature treatments created slow responses that were missed by our 40‐s threshold, a control for false‐negative results. After determining that mean response time did not differ by temperature (see Section 3), we used 40 s as the cutoff threshold to generate binary response/no‐response data for creating preference functions. The choice of 40 s as the threshold balanced the chances of missing slow responses against the possibility that nonresponsive females would make incidental contact with the speaker as they continued to walk within the arena.

#### 2.2.1 Describing mate preference functions

From the binary response data, we generated female preference functions and extracted peak, tolerance, and responsiveness (Figure 1) using a custom‐written R script, courtesy of J. Kilmer (script available upon request). For 11 of 69 functions, we were unable to resolve the function because the female did not respond at a given temperature (five females did not respond at one temperature, and three females did not respond at two temperatures). Consequently, we dropped 11 response functions from the analysis of peak and tolerance, but included them in the analyses of motivation. Of these eleven functions, three were generated at 30°C, three at 25°C, and five at 20°C.

We presented the entire stimulus set (11 playbacks per temperature category) to determine what range of pulse rates elicited female response. Individual preference functions were, however, calculated using trials that symmetrically bracketed the population peak at a given temperature. This is important for avoiding artifacts due to sampling intensity. Because we presented the same stimuli at all temperatures, the preference function peak fell closer to the high end of the stimulus set in the warm trials and closer to the low end of the stimulus set in the cool trials. As stimuli diverge from the preferred value, female preference drops, but even relatively distant values can receive occasional responses. When functions are generated from stimuli that are approximately centered on the response peak, these occasional responses and incidental contact have an equal possibility of occurring to stimuli on either side of the peak. However, when the preference peak falls nearer to one end of the stimulus set, there are relatively few stimuli on one side of the peak that could accumulate responses, but many stimuli on the other side, making the probability of any one of these receiving a response much higher and skewing the functions slightly toward whichever side of the peak was better sampled.

If the population peak matched one of the stimuli, we used this peak value and two stimuli on either side to construct the function. If the peak fell between two stimuli, we used the three stimuli on either side of the peak. The window selected was the broadest possible given the position of the population peak relative to the ends of the stimulus series. This window (at least 25 pulses/s) was broad relative to the observed variation in the male traits (*SD* ~3 pulses/s at 25°C).

#### 2.2.2 Describing overall motivation to search for mates

We obtained two measures of the overall motivation of female phonotaxis. First, we measured the time until the female made contact with the speaker broadcasting the stimulus at each of the three different temperatures (see above). Second, we assessed how temperature affected the motivation of females to approach the speaker by quantifying how many responses were recorded at each temperature, using the 40‐ and 120‐s time limits (see above).

### 2.3 Statistical analysis

We used linear regression to quantify intra‐ and interspecific effects of temperature on male signals. In these models, the pulse rate of the male signal was the dependent variable, with temperature, species, and temperature × species as fixed effects.

We then used linear mixed models in the software JMP (v.11, SAS Institute, Inc., Cary, NC, U.S.A.) to determine whether peak preference, tolerance, or responsiveness changed with temperature. We fit the linear mixed models with temperature as a fixed effect, and individual identity as a random effect to control for the fact that the same females were tested.

To assess the effect of temperature on the signal‐preference relationship, we compared the rate of change in signal pulse rate with the rate of change in peak preference. We used linear mixed models in which the dependent variable was pulse rate (of the male signal or of the peak of female preference function). The following were fixed explanatory variables: sex, temperature, and the sex × temperature interaction. The interaction term tests for differences in the slope of the relationship between temperature and signal pulse rate for males and peak preference for females—that is, it tests for temperature coupling. The model also included individual identity as a random effect to control for the fact that the same females were tested across all three temperatures.

To assess the effect of temperature on species discrimination, we used a generalized linear model (normal distribution, identity link function) to test how the rate of decrease in female preference tolerance compared with the rate of decrease in between‐species differences in signal pulse rate. The dependent variable in this model was the pulse rate difference (males) and tolerance (females), with sex, temperature, and the sex × temperature interaction as fixed explanatory variables. The interaction term tests for whether the slope of the temperature relationship in the two traits that are involved in species discrimination (heterospecific signal difference and tolerance of male signal variants) differs by sex. A significant interaction term would suggest that at low temperatures the narrower preference tolerance cannot make up for the smaller pulse rates differences between con‐ and heterospecific males.

We also assessed two measures of female motivation to approach male signals. To determine whether response time was affected by temperature, we fit a linear mixed model with temperature as a fixed effect, and individual identity as a random effect. To test whether the likelihood of response varied with temperature, we used binary response/no‐response data from each trial and fit a generalized linear model with binomial link function, including temperature as a fixed effect and individual and stimulus as random effects.

## 3 RESULTS

**Figure 5:**
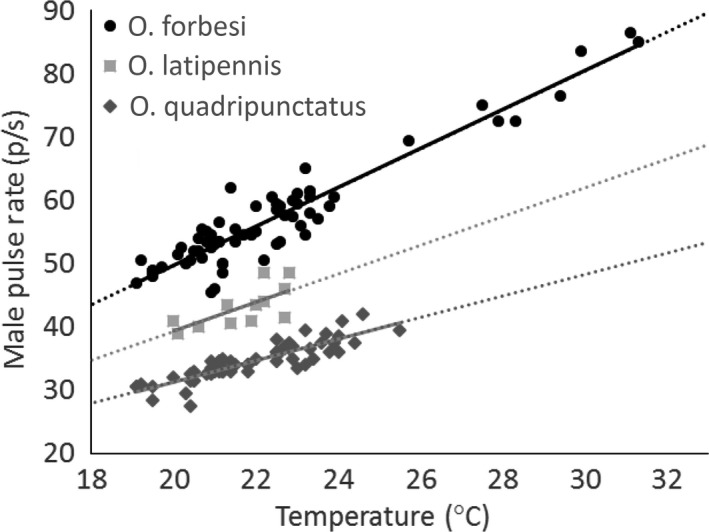
The pulse rate of male tree cricket signals increases with temperature. However, the rate of increase differs across species, with faster pulse rate species having greater increases in pulse rate with temperature (*Oecanthus forbesi* pulse rate = 3.08 × temp°C − 11.79, *R* ^2^ = .90; *Oecanthus latipennis* pulse rate = 2.26 × temp°C − 5.85 *R* ^2^ = .49; *Oecanthus quadripunctatus* = 1.70 × temp°C − 2.68, *R* ^2^ = .77). Consequently, heterospecific signals are more similar at cool temperatures (see also Figure 8a,b). Symbols represent recordings of individual males (*N*_forb_ = 66; *N*_lat_ = 13; *N*_quad_ = 66). Fitted lines are dotted where extrapolated to temperatures where individuals signal but were not recorded. All three species shown produce continuous pulsed acoustic signals and occur sympatrically and syntopically in western Ohio (USA)

In all three species of tree cricket, the pulse rate of male songs differed among species and increased with temperature. However, the rate at which pulse rates change with temperature varied across species, with the consequence that male signals of different species were more similar at lower temperatures (Table 1, Figure 5).

**Table 1:** ANOVA of male signal pulse rates in *Oecanthus* tree crickets, partitioning variation according to species identity, temperature, and the interaction between species and temperature. The interaction term captures interspecific differences in how pulse rate changes in response to temperature

	*df*	MS	*F*	*p*
Species	2	7353.8	1450.05	<.0001
Temp	1	482.7	95.2	<.0001
Species × Temp	2	102.8	20.3	<.0001
Error	138	5.07		

**Figure 6:**
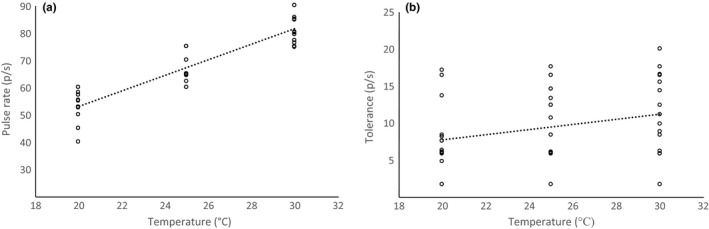
Effect of temperature on female preferences. The peak of the female pulse rate preference function increased with temperature (panel a). At cooler temperatures, females had lower tolerance for deviation in male traits (panel b). Each point represents the preference of one female at one temperature (*N* = 18 females per temperature)

The mate preferences of female *O. forbesi* tree crickets were also affected by temperature. Peak preference for pulse rate increased with temperature (*F*_1,39.5_ = 231.8, *p* < .0001, Figure 6a). Tolerance, that is, the range of signal values that elicit a given level of response, increased with temperature; that is, females were more discriminating at cool temperatures (*F*_1,55.0_ = 243.0, *p* < .0001, Figure 6b).

**Figure 7:**
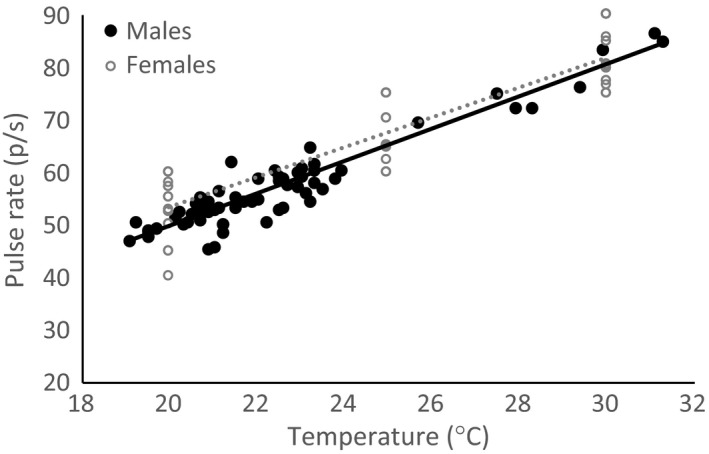
Relationship between the pulse rate of males (filled circles) and the peak preference of female (open circles) *Oecanthus forbesi* tree crickets. Male pulse rate and female peak pulse rate preference increased at similar rates (Female slope = 2.83 ± 0.19(p/s)/°C; male slope = 3.07 ± 0.13(p/s)/°C), resulting in temperature coupling. Filled symbols and solid line denote male signal pulse rates, and open symbols (one point per female per temperature) and dotted lines denote female preferences

**Figure 8:**
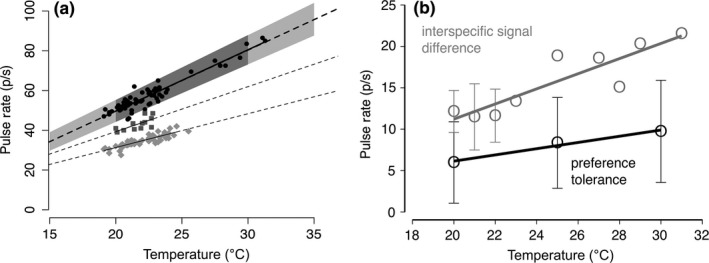
Although the tolerance window for pulse rates that females find acceptable did not overlap with the mean pulse rate of heterospecifics in the tested temperature range (comparison of shaded zone and mean heterospecific line in panel a), as temperatures drop further, the tolerance window increasingly overlaps with the fastest pulse rates of heterospecific individuals. Solid lines show mean pulse rates of male signals, and the shaded zone shows the range of pulse rates that elicit a robust response at a given temperature (tolerance). Dark shaded area and dark symbols show experimental data. Light shading and dotted lines show extrapolated data. Females respond to a narrower range of traits at cool temperatures (i.e., tolerance is lower), but because preference tolerance decreases more slowly than the pulse rate difference of male signals, the potential for interference actually increases at cool temperatures (panel b). Shown are mean values ± *SD*. For some temperatures (20–22°C), we had sufficient male recordings to calculate interspecific signal differences between actual male signals (hence, *SD* error bars); for other temperatures, sample size was lower and only allowed for calculating signal difference between individual *Oecanthus forbsei* recordings and the extrapolated *Oecanthus latipennis* mean signal pulse rate

The relationship between signal pulse rate and peak preference in *O. forbesi* remained constant across temperatures (i.e., there was temperature coupling)—the decrease with temperature in pulse rate matched the decrease in peak preference (Table 2, Figure 7). By contrast, preference tolerance decreased more slowly than the between‐species difference in signal pulse rate (Table 3, Figure 8a,b) (i.e., the effect of temperature on tolerance was detectable but small). Thus, the absolute and relative capacity for discrimination decreased at cooler temperatures. At the lower range of temperatures tested, the tolerance window did not overlap the mean difference between heterospecific signals, but it increasingly overlapped with the more extreme heterospecific signals (Figure 8b).

**Table 2:** ANOVA comparing the effect of temperature on male pulse rate and the peak of the female pulse rate preference function

	*df*	*F*	*p*
Sex	1, 90.4	10.5	.0017
Temp	1, 115	581.3	<.0001
Sex × Temp	1, 115	0.99	.3235

**Table 3:** Generalized linear model comparing the decrease with temperature in the species difference in signal pulse rate and in the tolerance for pulse rate of females

	*df*	χ^2^	*p*
Sex	1	21.71	<.0001
Temp	1	13.87	.0002
Sex × Temp	1	3.86	.049

By contrast, temperature had no effect on responsiveness, that is, the mean height of the female preference function (*F*_1,19.9_ = 1.21, *p* = .28). The motivation of females to perform phonotaxis was also not affected by temperature. Temperature had no effect on response times (*F*_1,363.1_ = 2.23, *p* = .14). Furthermore, temperature did not affect the number of stimuli that elicited response in under 40 s (*df* = 2, χ^2^ = 2.03, *p* = .36), or the number of stimuli that elicited response in under 120 s (*df* = 2, χ^2^ = 1.80, *p* = .41).

## 4 DISCUSSION

**Figure 9:**
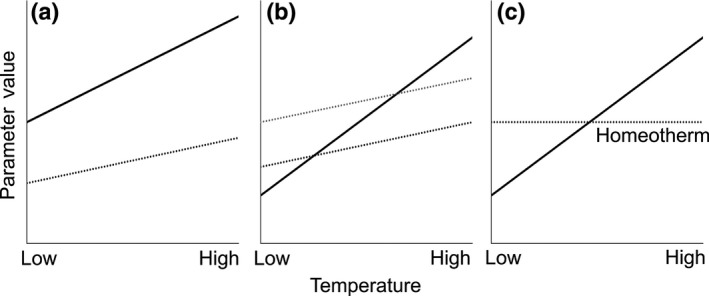
When species have different temperature dependence of signal parameters, the potential for interference will vary. (panel a) The species with the highest signal parameter value also has the steepest temperature dependence. In this scenario, temperature will affect signal similarity, but will not result in identical signal parameters. (panel b) When species with low parameter values have steep temperature dependence (black line), there will be a temperature where parameters of the signals will match the parameters of less‐temperature‐dependent heterospecifics (dotted line). If either or both species undergo character displacement as a result of reproductive interference, the temperature of maximum interference will change (arrows), but interference will still occur unless the slope evolves or displacement is sufficient to move the intersection out of the range of biologically relevant temperatures. (panel c) Interactions between heterotherms and homeotherms may be particularly challenging because the slope of the homeotherm temperature relationship is relatively flat

In this study, we adopted a community approach to ask how temperature affects signal similarity between three closely related *Oecanthus* species, and the relationship between male signals and female preferences in one of those species, *O. forbesi*. Male signal pulse rate and the peak of female preferences for pulse rate showed comparable temperature dependence. Additionally, we document that temperature can not only change peak preferences, but also impact tolerance for variation in male signals. When temperatures were cooler, females tolerated less deviation in signals. However, the difference between signals that females accept and heterospecific signals was not constant across all temperatures, a finding with potential consequences for interspecific interference and community dynamics (Figure 9).

### 4.1 Effect of temperature on signal interference

As temperatures cool, females tolerate less deviation from their peak preference. At the low end of the temperature range tested in our study, the tolerance window for pulse rates that females find acceptable did not yet overlap the mean heterospecific pulse rate, but increasingly overlapped with the fastest pulse rates of heterospecific individuals. If this pattern holds beyond the temperature range tested in our study, species discrimination may be jeopardized at low temperatures. The *Oecanthus* species included in this study have been recorded calling at temperatures as low as 13°C (Walker, 1963), although calling activity decreases at these cooler temperatures (L. Symes, per obs).

### 4.2 Effect of temperature variability on community composition

The spatial and temporal variability of temperature could have important consequences for the incidence of both signal interference and recognition errors. The testing conditions in this study represent a thermally stable environment, where the female is at a constant temperature. Natural environments can have substantial thermal heterogeneity (Gunderson & Leal, 2012; Logan, Fernandez, & Calsbeek, 2015; Logan, Huynh, Precious, & Calsbeek, 2013), even after dark when there is no longer an input of solar radiation. At 20°C, a difference of 2°C between male and female body temperature could offset the signal and preference peak by up to 6 pulses/s, enough to result in overlap between the female tolerance window and heterospecific signals. The acoustic signals of co‐occurring species are generally separated by a minimum of 8–10 pulses/s at 25°C (~5–6 pulses/s at 20°C) (Symes, 2014). If species with more similar signals experience fitness costs from signal interference, there would be a limited number of continuously signaling species that could partition signal space. Incorporating additional signal parameters such as chirps and trills may provide a way of generating perceptually unique signals.

Spatial variation in temperature is common, but the absolute variation in temperature may be more common at warm temperatures, particularly if solar radiation has warmed some areas more than others. Factors such as wind, humidity, vegetation, and topographic relief will all likely affect the extent of thermal heterogeneity. Environmental heterogeneity in temperature may have particularly dramatic consequences for communication of ectothermic organisms such as frogs that use both terrestrial and aquatic signaling environments. Air and water temperatures are often quite different, meaning that these organisms have access to a wide range of temperature environments, some of which are actively sought by organisms (Höbel & Barta, 2014).

### 4.3 Effect of between‐species variation in temperature dependence

Temperature effects on signal discrimination may occur in a broad variety of animals. While temperature dependence is best described for acoustic signals, there is often temperature dependence in signals that are substrate borne (Cocroft, Rodriguez, & Hunt, 2010; Shimizu & Barth, 1996), visual (Iguchi, 2010; Michaelidis, Demary, & Lewis, 2006), and electrical (Dunlapa, Smithb, & Yektaa, 2000). In taxa where females not only respond to acoustic signals of heterospecifics, but sometimes mate with heterospecifics after responding, the risk of interspecific mating may not be evenly distributed through space and time, but will concentrate at particular temperatures, geographic locations, or times of the year.

Across taxa, when species differ in the temperature dependence of their signal parameters, the potential for interference may span the gamut from little to severe interference (Figure 9). In some scenarios, temperature will affect signal similarity, but should not result in significant signal overlap (Figure 9a). But if temperature‐dependence functions of similar signals cross, the potential for interference becomes more acute. For example, one species may have a low trait value that increases sharply with temperature, while a second species has a slightly higher trait value but shallower temperature dependence, or essentially no temperature dependence in the case of homeotherms (Figure 9b,c). When the species with the lower intercept has steeper temperature dependence, it is inevitable that the signal characteristics of the two species will overlap at some temperature, potentially a temperature at which both species are active. When signal characteristics overlap, one or both taxa may undergo reproductive character displacement as a result of reproductive interference (Jang & Gerhardt, 2006; Kirschel et al., 2009). However, if the species with the steeper temperature‐dependence slope still has the lower intercept, the shift in traits will simply shift the temperature at which the two slopes intersect (Figure 9b). Consequently, even taxonomically distant species in a location may show similar temperature dependence, or at least correlation between slope and intercept. The effect of nonequivalent response to temperature may be particularly apparent when interference occurs between homeotherms and heterotherms because homeotherm signals are unlikely to vary with temperature, while the signals of heterotherms frequently do, meaning that there is always a temperature at which the heterotherm could encounter interference from the homeotherm (Figure 9c).

## 5 CONCLUSION

The way in which signals are perceived and processed has consequences for intraspecific mating preferences and interspecific signal interference. Environments vary in many ways including temperature, species composition, signal degradation, and predation intensity, all factors with the potential to interact with signaling, sexual selection, and mate recognition (Safran, Scordato, Symes, Rodríguez, & Mendelson, 2013; Scordato, Symes, Mendelson, & Safran, 2014). The findings of this study reveal how one environmental factor (temperature) affects male signals and female preferences. Further expansion of the taxonomic scope and environmental factors will provide insight into how ecological factors interact with the process of communication and mate choice in diverse communities.

## DATA ACCESSIBILITY

Male song recordings and signal measurements are archived at the Macaulay Library of Natural Sound (Cornell University). Specimens are archived at the California Academy of Natural Sciences.

## CONFLICT OF INTEREST

None declared.
